# Ecological and Socioeconomic Predictors of Transmission Assessment Survey Failure for Lymphatic Filariasis

**DOI:** 10.4269/ajtmh.18-0721

**Published:** 2019-05-20

**Authors:** Ellen M. Goldberg, Jonathan D. King, Denise Mupfasoni, Kevin Kwong, Simon I. Hay, David M. Pigott, Elizabeth A. Cromwell

**Affiliations:** 1 Institute for Health Metrics and Evaluation, University of Washington, Seattle, Washington;; 2 World Health Organization, Geneva, Switzerland

## Abstract

The transmission assessment survey (TAS) is recommended to determine whether cessation of mass drug administration (MDA) for lymphatic filariasis (LF) is warranted. Ministries of health typically implement TASs in evaluation units (EUs) that have had more than five rounds of annual MDA. Under TAS guidelines, sample size calculations determine a decision value: if the number of individuals testing positive exceeds this threshold, then MDA continues in the EU. The objective of this study was to determine whether fine scale geospatial covariates could be used to identify predictors of TAS failure. We geo-referenced 746 TAS EUs, of which 65 failed and extracted geospatial covariates using R to estimate odds of failure. We implemented stepwise backward elimination to select covariates for inclusion in a logistic regression to estimate the odds of TAS failure. Covariates included environmental predictors (aridity, distance to fresh water, elevation, and enhanced vegetation index), cumulative rounds of MDA, measures of urbanicity and access, LF species, and baseline prevalence. Presence of *Brugia* was significantly associated with TAS failure (odds ratio [OR]: 4.79, 95% CI: 2.52–9.07), as was population density (OR: 2.91, 95% CI: 1.06–7.98). The presence of nighttime lights was highly protective against failure (OR: 0.22, 95% CI: 0.10–0.50), as was an increase in elevation (OR: 0.36, 95% CI: 0.18–0.732). This work identifies predictors associated with TAS failure at the EU areal level, given the data presently available, and also identifies the need for more granular data to conduct a more robust assessment of these predictors.

## INTRODUCTION

Lymphatic filariasis (LF) is a mosquito-borne parasitic infection caused by *Wuchereria bancrofti*, *Brugia malayi*, and *Brugia timori*. Transmission among humans is largely sustained by mosquitos from the genera *Anopheles*, *Aedes*, *Culex*, and *Mansonia*, and typically observed in rural and underserved communities. *Wuchereria bancrofti* are responsible for 90% of human cases, and disease transmission occurs when a mosquito bites a human infected with LF, acquiring the microfilariae circulating in the blood. The microfilariae develop into larvae, which can enter a new human host when the infected mosquito is feeding. The mosquito-borne larvae then develop into thread-like adult-stage worms and can live 4–6 years in the human lymph system, producing microfilariae and continuing the transmission cycle.^1^

Prolonged and repeated infection can result in chronic physical manifestations that are a result of the general impact on the lymphatic system, characterized by swelling of the limbs (lymphedema) or scrotum (hydrocele).^2^ Episodes of acute disability occur generally in the form of adenolymphangitis (ADL), often a precursor to lymphedema involving fever, swelling, and malaise. Adenolymphangitis episodes can last for 3 to 15 days at a time, and recur multiple times in a year.^3^ Although most LF-endemic areas are found in Africa and Southeast Asia, the global distribution of LF is widespread, with evidence of either presently or previously endemic countries in the Caribbean, South America, the Middle East, and the Pacific Islands. Historical estimates suggest that approximately 119 million people may have been infected with LF globally and another 1.3 billion reside in areas suitable for transmission in 2000.^4,5^ The burden of LF-related disability has also been quantified, with more than 1.9 million disability-adjusted life years attributed to hydrocele, lymphedema, and ADL in 2000.^6^

In 1997, the World Health Assembly adopted resolution WHA50.29, the “elimination of lymphatic filariasis as a public health problem” followed by the launch of the Global Program to Eliminate LF (GPELF) in 2000.^7,8^ Under GPELF, the WHO recommends annual mass drug administration (MDA) with antihelminthic medicine (diethylcarbamazine [DEC], DEC and albendazole, or ivermection and albendazole) for at least five consecutive years, reaching a minimum of 65% of the population residing in endemic areas defined as implementation units (IUs), typically following second-order administrative boundaries such as districts or counties. The objectives of the GPELF were to interrupt transmission of infection and reduce the incidence of LF-related morbidity through the treatment of at least 845 million individuals globally for at least 5 years.^9^

From 2000 to 2009, more than 2.8 billion treatments were administered to the population at risk of infection, and in 2011, the WHO introduced the methodology for the transmission assessment survey (TAS) to determine whether IUs could cease MDA implementation and begin post-MDA surveillance.^9^ Under the TAS design, a sample of approximately 1,500 children ages 6–7 years from either communities or schools is tested for LF infection across an area of space defined as the evaluation unit (EU). Evaluation unit boundaries can be equivalent to IU boundaries, but can also be formed by combining multiple IUs into a single EU for survey or by subdividing an IU into multiple EUs. Species and dominant vector type, net primary-school enrollment ratio, the total number of 6- and 7-year-olds in an EU, and the number of primary schools or census enumeration areas (EAs) impact the survey design for a TAS. According to various thresholds for each of these factors as defined in the TAS methodology, sampling is through either a primary-school or EA-based survey, using either a systematic sample or a cluster survey of 6- and 7-year-olds.^10^ Regardless of the type of sampling implemented, the EU is the unit of reporting for TASs and the underlying cluster-level rarely become publicly available. Sample size calculations dictate a decision rule (called a “critical cutoff value”) for the number of children who test positive that would approximate the minimum prevalence at which LF transmission theoretically could be sustained. These thresholds are species and vector specific, based on models used to determine the impact of MDA on LF transmission.^10–12^ If the number of children who test positive exceeds this “critical cutoff value,” then the EU fails the TAS and MDA should continue. If an EU passes the TAS, then annual MDA is no longer recommended and TASs are repeated an additional two more times over a 5-year period as part of post-MDA surveillance.^10^

To date, 92% of EUs have passed the TAS, but 18 countries have experienced at least one failed TAS.^13^ The objective of this study was to determine whether environmental covariates summarized over the geographic boundaries of the EU were associated with an increased odds of failing a TAS. Because the results of TASs implemented at the EU level are areal data, it is possible that areal-level information could be used to characterize these units of space. If predictors of TAS failure could be identified at the EU level, then national program managers could use such information to enhance implementation and supervision of MDA interventions among corresponding IUs. Furthermore, such predictors could be used to intensify the program monitoring of areas at a higher risk of failure.

## METHODS

### Input data.

Transmission assessment surveys were implemented by ministries of health and reported to the WHO as part of monitoring guidelines for the Global Program to Eliminate LF. In this analysis, TAS failure was defined by the number of children who test positive for LF across an entire EU exceeding the “critical cutoff value.” Sample size and cutoff values were calculated using a lot quality assurance sampling method and could be determined using a survey sample builder.^10^ These values were designed to ensure that an EU has at least a 75% chance of passing the TAS if the true prevalence of LF is half the threshold, and no more than a 5% chance of falsely passing the TAS if the true prevalence is greater than or equal to the threshold. The thresholds are species and vector specific, and are described in Table 1. If the critical cutoff value was not reported in the original data, then the TAS was assumed to have failed if the proportion of children testing positive exceeded the theoretical prevalence thresholds as defined by LF species and vector. In this analysis, if both LF species were co-endemic, then the TAS was classified as a failure if the number of positive in a TAS exceeded at least one critical cutoff value. Children who tested positive using diagnostics, including the circulating antigen tests, rapid immunochromatographic test (ICT), and Filariasis Test Strip (FTS) and filarial antibody detection tests for *Brugia* spp., such as the Brugia Rapid™ test (Reszon Diagnostics International, Subang Java, Selangor, Malaysia), were considered to be positive for LF infection.^10^ Only the summary outcomes of the number of people tested and the number found positive were available for each TAS.

**Table 1:** Species- and vector-specific thresholds for transmission assessment surveys

Species type	Dominant vector	Threshold (%)
*Wuchereria bancrofti*	*Anopheles* and/or *Culex*	< 2
*W. bancrofti*	*Aedes*	< 1
*Brugia* spp.	All vectors	< 2

TAS = transmission assessment survey. In areas where *W. bancrofti* is endemic and *Anopheles* and/or *Culex* are the principal vectors, the threshold is less than 2%; less than 1% in areas where *W. bancrofti* is endemic and *Aedes* is the principal vector; and less than 2% in areas where *Brugia* spp. is endemic. These thresholds approximate the minimum prevalence at which lymphatic filariasis transmission theoretically could be sustained, and a transmission assessment survey fails if it exceeds the threshold.

#### Geo-referencing TAS EUs.

Geographic information for EUs and IUs were reviewed to first determine whether the geographic boundaries were equivalent to IUs for areas that implemented MDA. If so, they were geo-referenced to administrative boundaries by reviewing country maps and fuzzy-matching IU names to polygons in either GAUL or GADM shapefiles in ArcMap (10.4.1) (Environmental Systems Research Institute, Redlands, CA). If GAUL or GADM administrative boundaries did not reflect programmatic boundaries, then an IU shapefile maintained by the Expanded Special Project for Elimination of Neglected Tropical Diseases (ESPEN) was used for Africa or the NTD IU shapefile maintained by NTDMap.org for IUs outside of Africa.^14,15^ For EUs that covered multiple IUs, country program reports and published scientific literature were reviewed to create custom geography. If IUs followed standard administrative boundaries, then Global Administrative Unit Layer (GAUL) or Database of Global Administrative Areas (GADM) shapefiles were joined in ArcMap to create custom geographies. Two hundred thirty-one (31.0%) EUs comprised IUs that did not correspond to standard administrative boundaries, so program reports and publications in peer-reviewed journals were reviewed to identify maps of custom geographies which were then created in ArcMap 10.4.1.

#### Covariates.

Geo-referenced TAS data were linked to a range of environmental and socioeconomic covariates, selected after reviewing the literature for their potential to be associated with LF transmission or impact effectiveness of implementation.^16^ A summary is presented in Table 2 and more information on the source and definitions of these covariates is presented in the SI (Supplemental Table 1). We compiled these covariates as 5 × 5-km raster layers and extracted mean or maximum values over each EU associated with the geo-referenced TAS data. To extract geospatial covariates, we extracted every pixel value encompassed within each EU geography and calculated the respective summary statistic. These values are referred to as the EU mean.

**Table 2:** Summary of covariates used in logistic regression

Covariate	Description	Reference group	Summary statistic
Access	Travel time to nearest settlement of > 50,000 inhabitants	≤ 60 minutes	Mean
Aridity	Index from the climatic research unit time-series	≤ 1	Mean
Distance to rivers	Distance to rivers	≤ 25 km	Mean
Nighttime lights	Nighttime light index from 0 to 63	≤ 1.5	Mean
Elevation	Elevation measured in meters	≤ 200 m	Mean
EVI	Enhanced vegetation index	≤ 0.3	Mean
Irrigation	Mean percentage per pixel equipped for irrigation	≤ 5	Mean
Population density	Number of people per pixel	≤ 5,000 people per pixel	Mean
Species	Presence of *Wuchereria bancrofti* only versus presence of *Brugia* spp. or presence of *Brugia* spp. and *W. bancrofti*	*Bancrofti* only	Binary value
MDA	Maximum number of recorded rounds of mass drug administration	≤ 5 rounds of MDA	Maximum
Maximum baseline prevalence	Maximum baseline prevalence observed within the evaluation unit*	≤ 5% prevalence	Maximum

MDA = mass drug administration.

* Includes presence of microfilariae and antigenemia diagnostics.

**Figure 1.**
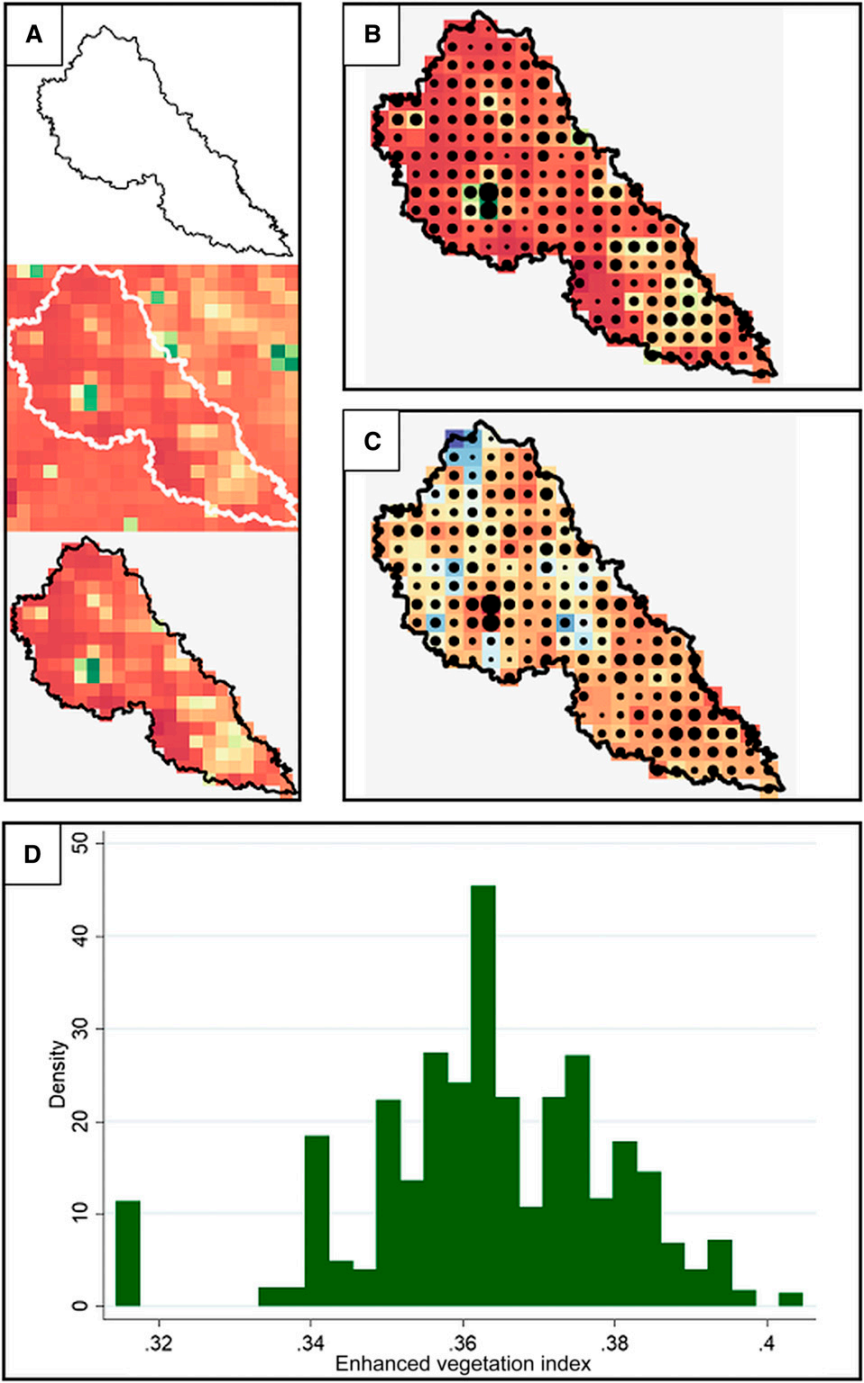
(**A**) An example EU shapefile (from Bangladesh) overlaid with a population density raster and cropped to the size of the shapefile.^28^ (**B**) Sampling of 1,000 pixels with replacement, weighted by population density. The size of the dot on each pixel represents the number of times it was sampled, with larger dots covering higher population density (green) areas. (**C**) Pixel-level draws overlaid with the enhanced vegetation index raster, and extracted to produce (**D**) a distribution of values over the EU, representing geographic heterogeneity. EU = evaluation unit. This figure appears in color at www.ajtmh.org.

To account for the variation in different covariate values across an entire EU, as well as avoid sampling locations without human habitation, we overlaid each EU geography with the population raster and sampled 1,000 pixels with replacement, weighted by population density.^17^ These 1,000 pixel-level values are referred to as pixel-level draws. We took these 1,000 sampled covariate values for each EU-year combination, matched by location (all covariate values were sampled from the same pixels), as illustrated in Figure 1. We then calculated the median value of these 1,000 population-weighted pixel-level values. These values are referred to as the EU draw median. Extraction of covariate values was performed using R version 3.4.3 (R Foundation for Statistical Computing, Vienna, Austria).

Continuous covariates were classified as binary categorical variables chosen to correspond to thresholds that could be translated into programmatic recommendations for identifying areas at a higher risk of failure. Reference groups were defined either by the distribution of extracted values or by previously determined limits for urbanicity and access found in prior studies.^18^

#### Exclusion criteria.

Because the TAS methodology was adopted in 2011, this analysis only includes TASs implemented from 2011 and onward reported to the WHO monitoring program or published in the peer-reviewed literature through September 2017. In addition, we excluded TAS observations implemented in historically non-endemic EUs for the purposes of confirmatory mapping, EUs that had incomplete data on the number of rounds of MDA, or EUs that were unable to be geo-referenced, and for TASs implemented among EUs defined as individual communities.

### Model.

#### Evaluation unit mean regression and pixel-level median regression.

We tested for an association between environmental and socioeconomic covariates and TAS failure using a logistic regression to estimate odds ratios (ORs). Using the non-population–weighted mean covariate values, we implemented backward elimination to select covariates for inclusion, with a *P*-value ≤ 0.15 for retention in the final model and a *P*-value ≤ 0.05 to determine statistical significance of associations.^19^ We also tested a logistic regression using generalized estimating equation (GEE) to account for dependency due to repeat measures within the same location as TASs are repeated up to three times for a single EU (e.g., TAS1, TAS2, and TAS3). Last, we conducted a sensitivity analysis to determine whether the thresholds used to create categorical variables biased our results, testing three different definitions for each independent variable, retaining the original covariate definitions as presented in the main analysis (see Supplemental Table 2a). We repeated these three regressions and sensitivity analysis for the pixel-level medians (see Supplemental Table 2b).

#### Evaluation unit pixel-level draws regression.

To simulate the uncertainty associated with aggregating covariate values over space, we ran 1,000 logistic regressions, one for each set of covariate values from the 1,000 pixel-level samples, each producing a beta coefficient and an estimated standard error. We then generated another 1,000 draws from each of the 1,000 distributions produced by those logistic regressions. This resulted in a total of 1,000,000 draws, representing the distribution of the association between environmental and socioeconomic covariates and TAS failure. We extracted the exponentiated mean, and 2.5th and 97.5th percentile values for each covariate, generating the mean and confidence intervals for the OR-associated covariates from the pixel-level draw simulation. Statistical analysis was conducted using STATA 13 (StataCorp LLC, College Station, TX).

## RESULTS

### Input data.

A total of 936 TAS records were reported to the WHO from 2011 to 2017 across 39 countries in South America, Africa, Asia, and Latin America. We excluded data that were unable to be geo-referenced (*N* = 108), and during the covariate extraction process, we were unable to extract all covariates across 10 additional TAS EUs because of small island geography and missingness in the covariate rasters, and so excluded these observations from the final dataset. Transmission assessment surveys implemented in EUs to confirm endemicity status or in areas otherwise considered non-endemic and never received MDA were also excluded (*N* = 32). We also excluded observations that had incomplete data on the number of rounds of MDA (less than a maximum of four rounds reported) (*N* = 40). A total of 75 TAS EUs were matched to IU boundaries using GAUL, 440 were matched using GADM, and for 231 EUs, the ESPEN or the other custom geography shapefile was used to match IUs. Three hundred forty-six observations were TAS1, 115 were TAS2 or repeated TAS1, and 45 were TAS3. We imputed whether a TAS passed or failed in seven observations using the population tested, the number of people who tested positive, and the species-specific thresholds.

Of the 746 total observations included in the analysis, 65 (8.7%) failed TAS, 531 (71.2%) were completed using ICT as the diagnostic tool, 125 (16.8%) used FTS, 63 (8.4%) used the Brugia Rapid test, and six (0.8%) used the identification of microfilariae in a blood smear; 59.1% of the total observations had a mean value of 60 minutes or less travel time to the nearest settlement of > 50,000 inhabitants, whereas 75.3% had a pixel-level median draw value of 60 minutes or less. Among the total observations, 43.0% had a mean value of an elevation more than 200 m and only 34.3% had a pixel-level median draw value of more than 200 m. Population density also differed significantly between the mean EU value and the pixel-level draw median, as 53.9% of observations had a mean value of more than 5,000 people per pixel and 61.3% had a pixel-level median draw value of more than 5,000 people per pixel. Only 17.4% recorded fewer than six rounds of MDA and 46.1% were located in areas endemic to *B.* spp. in addition to *W. bancrofti*. A summary of the covariate distribution for the analytical dataset is presented in Table 3.

**Table 3:** Characteristics of the TAS data and extracted geospatial covariates

	Evaluation unit mean, *N* (%)	Draw median, *N* (%)
TAS observations	746	
Pass*	681 (91.3)	
Fail	65 (8.7)	
Covariates		
Access		
≤ 60 minutes*	441 (59.1)	562 (75.3)
> 60 minutes	305 (40.9)	184 (24.7)
Aridity		
≤ 1*	375 (50.3)	382 (51.2)
> 1	371 (49.7)	364 (48.8)
Distance to rivers		
≤ 25 km*	274 (36.7)	293 (39.3)
> 25 km	472 (63.3)	453 (60.7)
Nighttime lights		
≤ 1.5*	373 (50.0)	411 (55.1)
> 1.5	373 (50.0)	335 (44.9)
Elevation		
≤ 200 m*	425 (57.0)	490 (65.7)
> 200 m	321 (43.0)	256 (34.3)
Enhanced vegetation index		
≤ 0.3*	249 (33.4)	259 (34.7)
> 0.3	497 (66.6)	487 (65.3)
Population density		
≤ 5,000 people per pixel*	344 (46.1)	289 (38.7)
> 5,000 people per pixel	402 (53.9)	457 (61.3)
Maximum baseline prevalence		
≤ 5%*	556 (74.5)	
> 5%	190 (25.5)	
MDA		
≤ 5 rounds*	130 (17.4)	
> 5 rounds	616 (82.6)	
Species		
*Wuchereria bancrofti* only*	402 (53.9)	
*Brugia* and *Brugia* + *W. bancrofti*	344 (46.1)	

MDA = mass drug administration; TAS = transmission assessment survey.

* Denotes reference value.

### Model.

#### Logistic regression: EU mean.

Nighttime lights, LF species type, elevation, maximum baseline prevalence, and population density were significantly associated with TAS failure (*P* < 0.05) in the logistic regression with backward elimination to select covariates for inclusion, using the mean values across each EU. Table 4 provides a summary of associations between the covariates and TAS failure for all models tested. These five predictors were statistically significant (*P* < 0.05) across the full and reduced logistic regressions, as well as in the GEE model, and the direction of their associations remained the same. Of the covariates that were retained in the reduced logistic regression, the presence of *Brugia* species was significantly associated with TAS failure (OR: 5.49, 95% CI: 2.84–10.62), as was population density of greater than 5,000 people per pixel (OR: 3.46, 95% CI: 1.24–9.64) and a maximum baseline prevalence of greater than 5% (OR: 2.38, 95% CI: 1.25–4.54). The presence of nighttime lights (greater than a mean index of 1.5) was highly protective against failure (OR: 0.21, 95% CI: 0.09–0.46), as was a mean elevation of greater than 200 m (OR: 0.36, 95% CI: 0.17–0.74). Distance to rivers and access were both retained in the reduced logistic regression, but were not statistically significant in the logistic or GEE regressions.

**Table 4:** Association between geospatial covariates and transmission assessment survey failure in a logistic regression with backward elimination covariate selection

Covariate	Logistic regression						
	EU mean			EU draw median			Pixel-level draws
	Full	Reduced*	GEE	Full	Reduced*	GEE	
	OR (95% CI)	OR (95% CI)	OR (95% CI)	OR (95% CI)	OR (95% CI)	OR (95% CI)	OR (95% CI)
Access	2.43 (0.88–6.70)	2.31 (0.84–6.35)	2.22 (0.76–6.43)	0.90 (0.41–1.99)	–	–	0.98 (0.34–2.78)
Aridity	0.60 (0.31–1.14)	–	–	0.52 (0.25–1.06)	0.54 (0.28–1.05)	0.58 (0.29–1.15)	0.62 (0.29–1.34)
Distance to rivers	1.78 (0.94–3.36)	1.76 (0.94–3.30)	1.64 (0.84–3.19)	1.28 (0.67–2.47)	–	–	1.50 (0.68–3.44)
Nighttime lights	0.21 (0.09–0.48)	0.21 (0.09–0.46)	0.22 (0.10–0.52)	0.08 (0.04–0.17)	0.08 (0.04–0.17)	0.09 (0.04–0.19)	0.22 (0.08–0.63)
Elevation	0.32 (0.16–0.65)	0.34 (0.17–0.68)	0.36 (0.17–0.74)	0.35 (0.15–0.80)	0.35 (0.16–0.80)	0.37 (0.16–0.85)	0.38 (0.13–0.99)
EVI	1.47 (0.71–3.02)	–	–	0.96 (0.46–2.01)	–	–	1.29 (0.53–3.10)
Population density	3.60 (1.28–10.13)	3.46 (1.24–9.64)	3.56 (1.22–10.37)	1.87 (0.87–4.03)	1.82 (0.94–3.50)	1.92 (0.97–3.81)	1.26 (0.53–3.10)
MDA	0.74 (0.37–1.48)	–	–	0.93 (0.45–1.93)	–	–	0.79 (0.38–1.65)
Maximum baseline prevalence	2.30 (1.20–4.42)	2.38 (1.25–4.54)	2.53 (1.27–5.03)	2.09 (1.07–4.07)	2.07 (1.07–4.02)	2.20 (1.10–4.39)	2.08 (1.04–4.20)
Species	6.04 (2.98–12.24)	5.49 (2.84–10.62)	5.68 (2.83–11.38)	7.28 (3.57–14.87)	7.36 (3.63–14.95)	7.27 (3.50–15.12)	5.39 (2.57–11.54)

EU = evaluation unit; GEE = generalized estimating equation; MDA = mass drug administration; OR = odds ratio.

* Backward selection used a *P*-value ≤ 0.15 for retention in the model.

#### Logistic regression: EU pixel-level draws.

When using the median pixel-level draw value for each TAS, nighttime lights, LF species, and elevation were again significantly associated with TAS failure (*P* < 0.05) in the logistic regression with backward elimination and in the GEE logistic regression. Aridity and population density were also retained, but only aridity was statistically significant in the reduced logistic regression, and was no longer statistically significant in the GEE logistic regression. The presence of *Brugia* species and a maximum baseline prevalence of greater than 5% were significantly associated with TAS failure in the reduced logistic regression (OR: 7.36, 95% CI: 3.63–14.95 and OR: 2.20, 95% CI: 1.10–4.39, respectively), whereas the presence of nighttime lights and mean elevation of greater than 200 m were both protective against failure (OR: 0.09, 95% CI: 0.04–0.19 and OR: 0.37, 95% CI: 0.16–0.85, respectively). The OR associated with the presence of nighttime lights decreased when compared with the estimates using the covariate means. The presence of *Brugia*, maximum baseline prevalence of greater than 5%, nighttime lights, and elevation greater than 200 m were all significantly associated with TAS failure in the GEE logistic regression.

In the aggregation of the 1,000 logistic regressions run on the 1,000 pixel-level draws, the presence of *Brugia* was significantly associated with TAS failure (OR: 5.39, 95% CI: 2.57–11.54) and a maximum baseline prevalence of greater than 5% was slightly associated (OR: 2.08, 95% CI: 1.04–4.20). Nighttime light was protective against failure (OR: 0.22, 95% CI: 0.08–0.63), and elevation greater than 200 m was slightly protective (OR: 0.38, 95% CI: 0.13–0.99). Table 3 shows a summary of associations and ORs for all model inputs and methods tested. Results of the sensitivity analysis are reported in the Supplemental Information.

## DISCUSSION

This study provides the first analysis on environmental and socioeconomic predictors of TAS failure. At this stage in the LF elimination program, the implementation of TASs across a range of geographic regions offers an opportunity to examine whether the areal classification of spatial covariates is associated with TAS failure. Our work identifies predictors that are associated with TAS failure at the EU areal level, given the data presently available, and also identifies the need for more granular data to conduct a more robust assessment of these predictors.

In this analysis, we sampled from pixel-level values to account for the potential heterogeneity of geospatial covariates that exist within EUs. In comparison to the logistic regression results without pixel-level sampling, the point estimates across all data inputs and methodological approaches do not vary significantly, which suggests that extreme variation in the covariate values did not contribute substantial bias. In addition, we used the GEE logistic regression on the mean and pixel-level median draw values so we could account for dependency between rounds of TASs conducted in the same EU. This also was not significantly different, suggesting that dependency due to repeated measures did not contribute much bias to the results, largely because of the few number of EUs with more than one observation.

The presence of nighttime lights is consistently and significantly protective of failure across a variety of models and data input types. We selected nighttime lights as a covariate as it can be indicative of socioeconomic factors, acting as a proxy for spatial distribution of quality living conditions, which has been shown to be protective against LF transmission.^20^ The OR for nighttime lights decreases when using the pixel-level median value and the covariate means across all models in the main and sensitivity analyses, likely because of the skewed distribution of nighttime lights and the difference between extracted mean and median values.

We also found that the presence of *Brugia* spp., in addition to *W. bancrofti* at the country level, is associated with failure. *Brugia* spp. differs from *W. bancrofti* with nonperiodic biting patterns and the presence of animal reservoirs, which could impact the effectiveness of intervention programs beyond MDA, such as insecticide-treated net coverage.^21^ In addition, the Brugia Rapid test identifies the presence of antibodies, which can indicate past, rather than current infection. As a result, this test is more sensitive, potentially increasing the likelihood of an EU failing a TAS where *Brugia* spp. are endemic. Elevation was shown to be protective, which is in agreement with previously published literature on disease transmission, which consistently finds that LF transmission is negatively associated with increasing elevation, likely because increased elevation is less suitable for vector survival.^16,22,23^

Maximum baseline prevalence of greater than 5% is also associated with failure across all regressions in the main analysis, suggesting that EUs with higher baseline prevalence may require more rounds of MDA to reach the elimination threshold. We attempted to leverage all available information to create this covariate, including geo-referenced values from published literature and early surveillance data. However, there were often very few or only one baseline observation per EU. In addition, we did not include an adjustment for varying age groups tested or diagnostic methods used, which included both the presence of microfilariae and antingenemia.

Population density was significantly associated with failure when using EU means, but was no longer significant when using the pixel-level draws or medians. It is often a predictor of LF transmission in some settings, and its fluctuating significance could be due to the different vector-specific transmission patterns, with *Culex* (primarily in East Africa and the Nile Delta) known for its urban transmission, and *Anopheles*, *Aedes*, and *Mansonia* for rural transmission.^16^ It is important to note the difference between the positive association of population density versus the negative association of nighttime lights and TAS failure. Whereas population density is typically greater where there is more urban development, the higher quality of living conditions and improved socioeconomic status is more protective against TAS failure than the impact population density has on LF transmission. The distribution of nighttime lights varies between TAS reported in Africa and the Asian setting, which may also result in an association with failure simply due to the distribution of the covariate in our dataset.

Although we have sought to leverage any available covariates in testing for predictors of TAS failure, we recognize a number of limitations in this analysis. We included LF species type, but were unable to include dominant vector classification in the regression because of incomplete vector data across all geographies. It is also possible that the association between *Brugia* species and TAS failure is a result of more failures generally reported from *Brugia*-endemic areas as many LF elimination programs in Asia and the Pacific began implementing TASs earlier than those in the African setting. Therefore, these locations contribute more failure events due to the programmatic timeline relative to when TAS guidelines were first introduced. LF is also different from other mosquito-borne diseases, such as malaria or dengue, in that its vectors belong to multiple genera, and experience a variety of breeding habitats and biting patterns, which can be influenced by both environmental factors and human-related activity.^24,25^ In addition, we were unable to account for insecticide-treated bednet coverage as this covariate is not available outside of Africa, which could have been indicative of the effectiveness of control and elimination efforts beyond MDA.^26^

Last, the TAS data are reported in aggregate for the EU. Characterizing geospatial variables over a large unit of space can be problematic and biased with varying amounts of heterogeneity and sensitive to any errors in geo-referencing. We try to account for this with the pixel-level population-weighted draws analysis, but leveraging the specific survey clusters from individual schools or communities would be necessary to better quantify the association between covariates and the presence of LF infection in children surveyed during TAS. Future analysis should examine the association between the number of children testing positive from the community or schools sampled during TAS using geospatial covariates extracted from those specific locations. If the number of children who test positive for LF infection clusters within a few locations across an EU, then such results could inform more targeted surveillance and monitoring at the sub-IU level.

Through the Global Program to Eliminate LF, 21 countries have completed LF interventions nationally and are undergoing surveillance, and an additional 25 countries have implemented the TAS and stopped MDA in at least one IU.^27^ Future research should examine predictors at a finer spatial scale than the EU, ideally to identify areas in need of enhanced monitoring before considering cessation of MDA. Detailed geospatial analysis to predict areas at risk of TAS failure could allow national programs to better target resources during this critical phase of the elimination program.

## Supplementary Files

Supplemental tables
