# Supplementary material for: Ecological and Socioeconomic Predictors of Transmission Assessment Survey Failure for Lymphatic Filariasis
Source: Am J Trop Med Hyg. 2019 May 20;101(1):271–8. doi: 10.4269/ajtmh.18-0721 (PMC6609191; doi:10.4269/ajtmh.18-0721)
Supplement: Supplementary file 1 [file tpmd180721.SD1.pdf]

Supplementary Information

Table S1: Covariate information and source

**Covariate**	**Temporal resolution**	**Source**	**Reference**
Access	Static	Malaria Atlas Project, University of Oxford	Weiss DJ, et al, 2018. A global map of travel time to cities to assess inequalities in accessibility in 2015. Nature 533: 333-336.
Aridity	Annual	Climatic Research Unit Time-Series (CRUTS)	Harris I, Jones PD, Osborn TJ, Lister DH, 2014. Updated high-resolution grids of monthly climatic observations – the CRU TS3.10 dataset. Int J Climatol 34: 623–642.<br><br>University of East Anglia. Climatic Research Unit TS v. 3.24 dataset. Available at: https://crudata.uea.ac.uk/cru/data/hrg/cru_ts_3.24.0 1/.
Distance to rivers	Static	Natural Earth Data (derived)	Natural Earth. Rivers and lake centerlines dataset. Available at: http://www.naturalearthdata.com/downloads/10mphysical-vectors/10m-rivers-lake-centerlines/.
Nighttime lights	Annual	Moderate Resolution Imaging Spectroradiometer (MODIS)	USGS & NASA. Land surface temperature and emissivity 8-day L3 global 1km MOD11A2 dataset. Available at: https://lpdaac.usgs.gov/dataset_discovery/modis/modis_products_table/mod11a2.
Elevation	Static	National Oceanic & Atmospheric Administration	National Centers for Environmental Information<br><br>Available at: https://www.ngdc.noaa.gov/mgg/topo/gltiles.html
Enhanced Vegetation Index (EVI)	Annual	MODIS	Huete A, Justice C, van Leeuwen W, 1999. MODIS vegetation index (MOD 13) algorithm theoretical basis document.<br><br>USGS & NASA. Vegetation indices 16-Day L3 global 500m MOD13A1 dataset. Available at: https://lpdaac.usgs.gov/dataset_discovery/modis/modis_products_table/mod13a1.<br><br>Weiss DJ, et al, 2014. An effective approach for gapfilling continental scale remotely sensed timeseries. Isprs J Photogramm Remote Sens 98: 106–118.
Population density	Annual	WorldPop	Lloyd CT, Sorichetta A, Tatem AJ, 2017. High resolution global gridded data for use in population studies. Sci. Data 4, sdata20171. World Pop. Get data. Available at: http://www.worldpop.org.uk/data/get_data/.
Species	Static	World Health Organization	Global Program to Eliminate Lymphatic Filariasis

		(WHO)	
Maximum rounds of Mass Drug Administration (MDA)	Annual	WHO	Global Program to Eliminate Lymphatic Filariasis
Maximum baseline prevalence	Static	WHO	Global Program to Eliminate Lymphatic Filariasis

Results of Sensitivity analysis

To determine how sensitive our findings were to the thresholds used to create categorical variables from continuous values extracted from geospatial covariates, we tested three separate categorical thresholds for each independent variable within the reduced logistic regression, using covariates as defined in the main analysis (Table S2 in the SI). Access and population density, covariates with thresholds defined through previously published literature, did not vary in this sensitivity analysis.

When using covariate means as the model input, species type and nighttime lights remained predictive in every variation of the model and the directionality of their effects did not change. Elevation and maximum baseline prevalence were retained in every iteration and statistically significant in every iteration except for one, and population density was included and statistically significant in 11 of the 12 models tested. Distance to rivers was included in 10 iterations, access was included in 9, while aridity was included in three. All three of these covariates were only statistically significant in one iteration each, and MDA was not included in any of the 12 models tested. When using the median pixel-level draw value, nighttime lights and species type remained significantly protective and predictive of failure, respectively, in every variation. Elevation, maximum baseline prevalence, and population density were retained in all but one iteration and were significantly protective against failure in 11, and predictive of failure in 11 and 3, respectively. Aridity was retained in 9 models and statistically significant in 3, while access, distance to rivers, EVI, and MDA were excluded from all 12 models tested. Table S2 in the SI provides a summary of associations between covariates and TAS failure for each iteration of this sensitivity analysis.

To address the potential bias introduced with the population-weighted sampling of pixels, we tested the pixel-level analysis without the population weighting but excluded all pixels that had a population density of fewer than ten people per 1 x 1 km and were classified as “barren or sparsely vegetated”.[17] Presence of nighttime lights was statistically significant and protective while population

density, species type, and maximum baseline prevalence were predictive of TAS failure across the full, reduced, GEE, and pixel-level logistic regression analyses. Increased elevation and aridity were statistically significant and protective against failure in the full, reduced, and GEE logistic regressions, but was no longer statistically significant at the pixel-level analysis. Table S3 in the SI provides a full summary of associations across the models tested.

We also tested the sensitivity of our exclusion of observations with incomplete MDA data (excluding surveys done in EUs with fewer than 5 rounds of recorded MDA) in the reduced logistic regression. Presence of *Brugia*, nighttime lights, maximum baseline prevalence, population density, and elevation were all retained and statistically significant, maintaining the same directionality as in the primary analysis. Additionally, we reran the primary analysis using effective MDA rounds only, meaning rounds with ≥ 65% coverage. When this covariate replaced the MDA covariate in our primary analysis, it was eliminated from the stepwise logistic regressions of the mean and median draw values, and failed to be significant in the full logistic regressions of both the mean and median draw value.

We also tested EU geographic size by extracting kilometers squared. When using a cutoff value of 2,500 km^2 (where 364 of 746 TAS observations were the reference value of ≤ 2,500 km^2), this was not significant in the original stepwise or simple logistic regression of the means. Similarly to the sensitivity analyses described above, we tested two other cutoff values (1,000 km^2 and 6,000 km^2) – only the latter was included in the stepwise analysis, and even then it was not statistically significant. We also tested EU size using total population in each EU using a cutoff value of 500,000 people, where 322 of 746 TAS observations were the reference value of ≤ 500,000 people. When using a cutoff value of 500,000 and 250,000, total population in each EU was not significant. However, when tested with a cutoff value of 2,000,000 people, it was slightly indicative of failures in the stepwise model. This is already a component of the TAS guidelines suggested by the WHO, and suggests that an EU defined over too large of an area may be less likely to pass TAS.

Table 2a: Reduced logistic regression using EU means with varying categorical thresholds

	Original stepwise	Aridity		Distance to rivers		Nighttime lights		Elevation		EVI		Max baseline prevalence	
		≤ 0.8	≤ 1.3	≤ 15 km	≤ 40 km	≤ 1	≤ 5	≤ 150	≤ 500	≤ 0.25	≤ 0.5	≤ 2%	≤ 10%
Access	2.15 (0.78 - 5.93)	2.15 (0.78 - 5.93)	2.15 (0.78 - 5.93)	--	--	2.21 (1.19 - 4.09)	2.21 (0.89 - 5.47)	--	--	2.15 (0.78 - 5.93)	--	2.41 (0.88 - 6.59)	2.15 (0.78 - 5.93)
Aridity	--	--	--	--	--	--	0.43 (0.22 - 0.82)	--	--	--	0.6 (0.32 - 1.15)	--	--
Distance to rivers	1.66 (0.91 - 3.07)	1.66 (0.91 - 3.07)	1.66 (0.91 - 3.07)	--	--	--	2.84 (1.40 - 5.78)	1.63 (0.88 - 3.03)	--	1.66 (0.91 - 3.07)	--	1.65 (0.89 - 3.06)	1.66 (0.91 - 3.07)
Nighttime lights	0.22 (0.10 - 0.50)	0.22 (0.10 - 0.50)	0.22 (0.10 - 0.50)	0.27 (0.15 - 0.49)	0.27 (0.15 - 0.49)	--	0.04 (0.02 - 0.10)	0.20 (0.09 - 0.44)	0.22 (0.10 - 0.48)	0.22 (0.10 - 0.50)	0.17 (0.08 - 0.39)	0.23 (0.10 - 0.50)	0.22 (0.10 - 0.50)
Elevation	0.36 (0.18 - 0.72)	0.36 (0.18 - 0.72)	0.36 (0.18 - 0.72)	0.39 (0.20 - 0.73)	0.39 (0.20 - 0.73)	0.43 (0.22 - 0.83)	0.25 (0.12 - 0.51)	0.53 (0.30 - 0.95)	0.51 (0.22 - 1.14)	0.36 (0.18 - 0.72)	0.41 (0.21 - 0.80)	0.33 (0.16 - 0.68)	0.36 (0.18 - 0.72)
Enhanced Vegetation Index	--	--	--	--	--	1.74 (1.19 - 4.09)	--	--	--	--	2.16 (0.98 - 4.77)	--	--
Population density	2.91 (1.06 - 7.98)	2.91 (1.06 - 7.98)	2.91 (1.06 - 7.98)	--	--	--	3.84 (1.52 - 9.71)	2.03 (0.95 - 4.35)	1.97 (0.94 - 4.09)	2.91 (1.06 - 7.98)	2.18 (0.94 - 5.08)	2.97 (1.09 - 8.13)	2.91 (1.06 - 7.98)
MDA	--	--	--	--	--	--	--	--	--	--	--	--	--
Max baseline prevalence	--	--	--	--	--	--	1.71 (0.87 - 3.34)	--	--	--	--	2.26 (1.29 - 3.94)	--
Species	4.79 (2.52 - 9.07)	4.79 (2.52 - 9.07)	4.79 (2.52 - 9.07)	4.83 (2.57 - 9.07)	4.83 (2.57 - 9.07)	3.45 (1.88 - 6.34)	8.85 (4.41 - 17.75)	5.17 (2.73 - 9.77)	5.02 (2.63 - 9.58)	4.79 (2.52 - 9.07)	4.95 (2.49 - 9.82)	5.69 (2.90 - 11.18)	4.79 (2.52 - 9.07)

The values in the headers are the alternative categorical thresholds for each independent variable within the reduced logistic regression. When a different threshold is tested for a single covariate, all other covariates remain constant, using the thresholds described in Table 3 of the manuscript.

Table 2b: Reduced logistic regression using EU pixel-level median draws with varying categorical thresholds

	Original stepwise	Aridity ≤ 0.8	Aridity ≤ 1.3	Distance to rivers ≤ 15 km	Distance to rivers ≤ 40 km	Nighttime lights ≤ 1	Nighttime lights ≤ 5	Elevation ≤ 100	Elevation ≤ 400	EVI ≤ 0.25	EVI ≤ 0.5	Max baseline prevalence ≤ 2%	Max baseline prevalence ≤ 10%
Access	--	--	--	--	--	--	--	--	--	--	--	--	--
Aridity	0.53 (0.28 - 0.99)	--	0.53 (0.27 - 0.99)	--	0.53 (0.28 - 0.99)	0.53 (0.28 - 0.99)	0.47 (0.25 - 0.90)	0.53 (0.28 - 1.02)	--	0.53 (0.28 - 0.99)	0.53 (0.28 - 0.99)	0.54 (0.28 - 1.03)	0.53 (0.28 - 0.99)
Distance to rivers	--	--	--	--	--	--	--	--	--	--	--	--	--
Nighttime lights	0.08 (0.04 - 0.17)	0.10 (0.05 - 0.20)	0.08 (0.04 - 0.17)	0.16 (0.08 - 0.33)	0.08 (0.04 - 0.17)	0.08 (0.04 - 0.17)	0.05 (0.02 - 0.12)	0.07 (0.03 - 0.15)	0.09 (0.05 - 0.19)	0.08 (0.04 - 0.17)	0.08 (0.04 - 0.17)	0.07 (0.03 - 0.16)	0.08 (0.04 - 0.17)
Elevation	0.37 (0.91 - 3.29)	0.47 (0.22 - 1.01)	0.38 (0.17 - 0.84)	0.41 (0.19 - 0.89)	0.37 (0.17 - 0.83)	0.37 (0.17 - 0.83)	0.39 (0.17 - 0.90)	0.50 (0.26 - 0.95)	--	0.37 (0.17 - 0.83)	0.37 (0.17 - 0.83)	0.36 (0.16 - 0.81)	0.37 (0.17 - 0.83)
Enhanced Vegetation Index	--	--	--	--	--	--	--	--	--	--	--	--	--
Population density	--	1.86 (0.98 - 3.52)	1.69 (0.88 - 3.22)	--	1.73 (0.91 - 3.29)	1.73 (0.91 - 3.29)	1.61 (0.86 - 3.03)	1.86 (0.98 - 3.53)	2.21 (1.19 - 4.09)	1.73 (0.91 - 3.29)	1.73 (0.91 - 3.29)	1.76 (0.91 - 3.41)	1.73 (0.91 - 3.29)
MDA	--	--	--	--	--	--	--	--	--	--	--	--	--
Max baseline prevalence	--	--	--	1.77 (0.90 - 3.48)	--	--	1.77 (0.91 - 3.42)	--	--	--	--	2.34 (1.21 - 4.19)	--
Species	6.88 (3.44 - 13.73)	5.81 (3.02 - 11.18)	7.04 (3.52 - 14.07)	4.70 (2.34 - 9.43)	6.88 (3.44 - 13.73)	6.88 (3.44 - 13.73)	8.22 (4.10 - 16.49)	8.10 (4.10 - 16.01)	7.41 (3.99 - 13.77)	6.88 (3.44 - 13.73)	6.88 (3.44 - 13.73)	5.61 (2.90 - 10.84)	6.88 (3.44 - 13.73)

The values in the headers are the alternative categorical thresholds for each independent variable within the reduced logistic regression. When a different threshold is tested for a single covariate, all other covariates remain constant, using the thresholds described in Table 3 of the manuscript.

Table S3: Association between geospatial covariates and transmission assessment survey failure in a logistic regression with backwards elimination covariate selection using non-population-weighted pixel-level draws

**Covariate**	Full<br>OR (95% CI)	Reduced*<br>OR (95% CI)	GEE<br>OR (95% CI)	Pixel-level Draws<br>OR (95% CI)
Access	1.09 (0.49 - 2.45)	--	--	1.05 (0.38 - 2.92)
Aridity	0.40 (0.20 - 0.82)	0.42 (0.22 - 0.83)	0.44 (0.22 - 0.87)	0.62 (0.28 - 1.40)
Distance to rivers	1.80 (0.91 - 3.56)	1.84 (0.95 - 3.57)	1.85 (0.94 - 3.67)	1.66 (0.73 - 3.98)
Nighttime lights	0.04 (0.02 - 0.10)	0.04 (0.02 - 0.11)	0.04 (0.02 - 0.11)	0.18 (0.05 - 0.57)
Elevation	0.38 (0.17 - 0.85)	0.38 (0.17 - 0.82)	0.38 (0.17 - 0.84)	0.37 (0.14 - 0.94)
Enhanced vegetation index	1.41 (0.64 - 3.10)	--	--	1.21 (0.49 - 3.13)
Population density	2.85 (1.16 - 6.99)	2.46 (1.21 - 5.03)	2.56 (1.23 - 3.67)	1.52 (0.62 - 3.91)
MDA	1.10 (0.54 - 2.26)	--	--	0.90 (0.43 - 1.88)
Max baseline prevalence	1.62 (0.83 - 3.17)	--	--	1.46 (0.75 - 2.86)
Species	9.05 (4.49 - 18.24)	8.25 (4.19 - 16.24)	8.13 (4.08 - 16.22)	5.30 (2.56 - 11.18)

* Backwards selection used a p-value ≤ 0.15 for retention in the model
